# Why Did America Declare War?

**Published:** 1918-08-10

**Authors:** 


### WHY DID AMERICA DECLARE WAR?

### ADDRESS OF DR. SHERWOOD EDDY. (*See p.* 409.)

At last Britain and America stand side by side in this great world struggle facing the common foe of humanity. Divided as America was in population, with some thirteen millions foreign born and eight millions of German descent, it took nearly three years to fuse that great isolated democracy into one burning unit of indignation, but at last we are united and stand with Britain to fight for a new and better world.

Why did America declare war?

With her own territory not invaded nor immediately threatened, her own trade not impaired but rather enhanced, with nothing to gain materially and everything to lose, why was America forced to fight? It was the growing menace of Prussian militarism; it was the challenge of a premeditated war of world conquest. The deliberate violation of Belgium, with the atrocities and barbarities that followed the devastation of Poland, with more than two millions already dead of starvation and disease under Prussian " organisation "; the systematic extermination of the Armenians; the sinking of the *Lusitania* and more than eight hundred neutral ships, before America declared war; the violation of American rights and property; the spreading network of the Prussian spy system, with its combination of cunning and violence; and lastly, when she was ready to strike the last fatal blow, the indiscriminate submarine warfare of Germany—these were the ten reasons which forced America to fight.

Coming in as late as we did, we can never forget the great price that Britain has paid and the noble sacrifice she has made for the whole world. For us, boasting is for ever excluded, and nothing that we can now do can make up for the time that has been lost; but we have done our best in a few brief months. Ten millions of men from twenty-one to thirty-one years of age were enrolled by conscription. Then the ships had to be built to carry the men, the munitions, and the food to the Allies. America, with half a million tons of ocean shipping, was only carrying one-tenth of her own foreign trade before the war. She now has fourteen hundred ships and fourteen times the ocean tonnage with which she entered the war. More than eight hundred ways when in full operation will soon be turning out six new ships a day. Munition works larger than Krupps are being built. The powerful Liberty motor for aeroplanes is being turned out in immense quantities. More money was spent and appropriated in the first year of the war by the States than Germany spent in the first three years of the conflict, and more than America spent in the first hundred years of her existence as a nation.

Standing at last together, in the face of the common foe, what are we fighting for? In a word, we are fighting together for Liberty, for Democracy, and for Right—Right as the basis of a lasting peace, Right as the ground of Government with the consent of the Governed. In a word, we fight for a new world—a world redeemed at great cost.

We fight to make the world worth their having died for, who have already made the great sacrifice for us all.

We fight for a new Internationalism, a League of Nations, as the basis of a lasting peace. We fight with a new faith in the common man, a new faith in the rank and file of Nations, the smaller as well as the larger, the weak as well as the strong, the backward peoples as well as the more advanced. We fight together for a new social justice, for new and better conditions of Labour. Let us not forget that Labour has given the most of the fighters in this war, and they rightly demand a juster world. We have learned during this war a new social control. In America the food supply, the railways, the telegraph and telephone, the entire coal supply and the shipping of the Nation have come under the control of the Nation. Never again must they be exploited for special privilege. As your own Premier has said, " This is everybody's world."

We are fighting in America for a new moral righteousness. We are fighting against drink and immorality. Twenty-seven States have already gone dry by an overwhelming majority by the free vote of the people. Within a year we shall have nation-wide Prohibition at the demand of the people themselves. We have tried to clean up our streets and to make the army and the country safe for our soldiers. We must not only win the war, but we must gain the great objects for which we are fighting.

Once again the great Anglo-Saxon race is united, the two nations (Britain and America) standing at last together. It is a solemn thought, sobering in its responsibility, to realise that together we control about one-third of the habitable land area of the globe. Together we have about one-third of the population of the world. The trade of the two nations combined forms about 60 per cent., or nearly two-thirds of the world's trade. Their combined wealth is greater than all the other nations that are fighting in the war—greater, in fact, than all the other nations of the world combined. But far more important than land or population, than trade or commerce or wealth, is the great heritage of our past, the common principles for which we stand. Together we have stood for Liberty, for Democracy, for Justice, for Righteousness. Let us not lower our ideals. We need no " hymn of hate " to make us fight. We are fighting to win the war, to win a new and better world, to win the high objects and ideals for which we will spend our blood, our might, and our treasure. And God will give the victory to the side that is in the right.

